# Delay-Differential-Equation Modeling of Mode-Locked Vertical-External-Cavity Surface-Emitting Lasers in Different Cavity Configurations

**DOI:** 10.3390/ma12193224

**Published:** 2019-10-01

**Authors:** Eugene A. Avrutin, Krassimir Panajotov

**Affiliations:** 1 Department of Electronic Engineering, University of York, York YO10 5DD, UK; eugene.avrutin@york.ac.uk; 2 Faculty of Engineering Sciences, Brussels Photonics Team B-PHOT, Vrije Universiteit Brussel, 1050 Brussels, Belgium; 3 G. Nadjakov Institute of Solid State Physics, Bulgarian Academy of Sciences, 1784 Sofia, Bulgaria

**Keywords:** vertical-external-cavity, surface-emitting lasers, mode locking, SESAM, frequency combs

## Abstract

A simple, versatile model for the dynamics of electrically and optically pumped vertical-external-cavity surface-emitting lasers, which are mode locked by a semiconductor saturable-absorber mirror, is presented. The difference between the laser operation in the linear and folded cavity, as well as the potential for a colliding pulse operation, are studied.

## 1. Introduction

Vertical-external-cavity surface-emitting lasers (VECSELs) were first developed in 1997 [1] and have the advantage of the mature VECSEL technology for the semiconductor gain chip, which consists of an epitaxial distributed bragg reflector (DBR) and several quantum well (QW) or quantum dot layers. This allows for wavelength flexibility, output power scalability, and mass production. The external cavity makes it possible for lasing to occur in a single transverse and longitudinal mode by implementing, respectively, spherical mirrors and Fabry–Perot filters in the external cavity [1,2,3,4,5]. On the contrary, multi-transverse mode lasing is typical for VECSELs with an output power in excess of several mW, due to spatial hole burning [6,7]. The spectral coverage of VECSELs extends from 390 nm [8] to 5 μm [9], and even to 244 nm [10], via an intracavity fourth harmonic generation. By efficient thermal management, the output power is scaled by simply scaling the area of the optically pumped spot, reaching 100W in Continuous Wave (CW) operation [11]. VECSELs are very well-suited for mode-locked operations by utilizing a semiconductor saturable-absorber mirror (SESAM), either in the external cavity [12], or as integrated into the gain chip [13]. Tremendous progress has been achieved since the first demonstration of an SESAM mode-locked VECSEL in 2000 [14]: The pulse durations in the fundamentally mode-locked operation have been decreased to 107 fs [15], and even to 60fs in a burst operation [16], while the average output power has been increased to 6.4W [17] and the peak power has been increased to 4.35 kW [18]. While the shortest pulses have been achieved with optically pumped active layers, electrically pumped structures are promising for a number of applications. A versatile, however fairly complex theoretical model has been presented [19,20] for mode locking in electrically pumped VECSELs, yet only for the simplest linear geometry, and using a semi-microscopic model for the optical properties of quantum-well active layers. Later, a delay-differential model of a similar type, yet with a simpler gain model, was used for the inclusion of complex transverse/lateral effects in a so-called MIXCEL structure, in which the active layer and the saturable absorber are hosted within the same chip [21]. This allows a possibility to substantially enhance computational efficiency, by narrowing the temporal window when simulating the pronounced Mode Locking (ML) regime [22]. In recent papers [23,24], a version of a delay-differential model was developed taking into account the folded, as well as simple linear, cavity geometries. These papers centred on lasers designed for ultrashort pulse generation, and were mainly dedicated to the issue of multiple pulse (pulse molecule) generation as observed under some operating conditions in such lasers; therefore the gain chip and saturable absorber were treated essentially as short travelling-wave amplifiers, ignoring their reflective nature and resonator properties (indeed, for femtosecond pulse generation it is beneficial to suppress the resonator properties of the chips by applying antireflection coatings, to minimise any narrowing of the emission spectra).

An alternative to the delay-differential equation modelling is an iterative pulse shaping approach [25,26,27], where gain, saturable absorption, and dispersion in each round-trip are, as in classic mode-locking theories, represented by pulse shaping operators in time or frequency domain, as appropriate. Such an approach is by necessity somewhat artificial, as it separates dispersion from gain and absorption, and also effectively assumes a unilateral ring cavity. Still, it has proven very useful for many cases of practical significance, particularly when the main limitation for the pulse duration is the group velocity dispersion of the cavity (soliton mode-locking regime). With simulation parameters deduced from the measurements for a known laser, such a model is capable of providing very good agreement with experiments in both picosecond [26] and femtosecond [27] regimes; however, it may not be the most appropriate model for describing regimes and designs where more than one pulse can exist in the cavity, or for including the cavity parameters at the design stage.

Here we continue the work started in the earlier conference papers [28], and present a model based on an approach similar to that of [19,20] and so treating the cavity properties of the gain and absorber chip consistently, by using a simpler, generic active layer model that can be used for both linear and folded-cavity geometry. The model can be, and is in this study, applied to study different regimes of laser operation, including the possibility of both colliding pulse and multiple colliding pulse mode locking, and which can be used for the inclusion of polarization effects in future.

The paper is organized as follows. In Section 2 we present the derivation of the model and its application to a straightforward linear cavity. In Section 2 and Section 3 we deal with different versions of a folded cavity, with either the gain or the absorber forming the central chip. Finally, in Section 4, a brief discussion and summary are presented.

## 2. Vertical-External-Cavity Surface-Emitting Laser: Time-Delay Model

### 2.1. Derivation of a Simple Equation for the Active Cavity Dynamics

**Figure 1:**
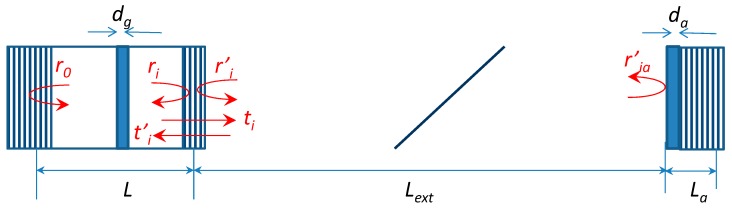
Mode-locked vertical-external-cavity surface-emitting laser (VECSEL) consisting of a vertical-cavity amplifier chip with two distributed bragg reflectors (DBRs), with reflectivities r_0_ and r_1_ and an active region with thickness d_g,_ and a semiconductor saturable-absorber mirror (SESAM). chip with a single DBR and the active region thickness d_a_. L and L_a_ are their effective lengths, and L_ext_ is the length of the external cavity.

A schematic of the mode-locked VECSEL consisting of vertical-cavity amplifier chip (left side) and a SESAM chip (right side) is shown in Figure 1.

The derivation of the model is a somewhat simplified version of that of [19], and is shown here for completeness. As in [19], we start with the frequency domain approach and then convert it into the time domain. The equation for the reflected “field” (more accurately, wave amplitude) leaving the gain chip reads:

(1) $$E_{r}^{\left(g\right)}=r_{g}E_{inc}=\left(\frac{t_{i}^{2}r_{o}\tilde{G}e^{-j2\tilde{k}L}}{1-r_{i}r_{o}\tilde{G}e^{-j2\tilde{k}L}}+r_{i}{}^{\prime }\right)E_{inc}=E_{rc}+r_{i}{}^{\prime }E_{inc},$$

In this equation:

*E_inc_* is the complex amplitude of the incident field.

*E_rc_* is the complex amplitude of the field exiting the active resonator chip into the passive compound cavity.

*r_i_* and *r_o_* are the (wavelength-dependent) reflectances of the mirrors of the resonator facing inside (i.e., the incident light), and outside the cavity *r_i_′* is the reflectance seen by the light incident on the mirror from the external cavity side, which has the same amplitude as *r_i_*, however, with a different phase, as usual.

*L* is the geometric cavity length.

$\tilde{k}$ is the complex wave vector. We can define a reference frequency and the corresponding wave vector $k_{ref}=\frac{n}{c}\omega_{ref}$ whereby, for convenience, it is easiest to assume that $\omega_{ref}$ is the frequency of one of the cold cavity modes. Then, $\tilde{k}=k_{ref}+\frac{n}{c}\Delta \omega -\frac{j}{2}\alpha_{\mathrm{int}}$, $\alpha_{\mathrm{int}}$ being the internal loss in the passive part of the resonator.

$\tilde{G}$ is the single-pass dimensionless complex gain by all the QWs in the active layer of the resonator. Assigning the active layer a thickness *L_a,_* and introducing the equivalent distributed complex gain $\tilde{g}=g{}^{\prime }+jg{}^{\prime \prime }$ we can write $\tilde{G}=e^{\xi_{g}\Gamma_{⊥}\tilde{g}d_{g}}=e^{\Gamma \tilde{g}L}$, where the total confinement factor, including the enhancement, or relative confinement, factor due to the standing wave profile *ξ_g_* is:

(2) $$\Gamma =\xi_{g}\;\Gamma_{⊥}d_{g}/L$$

This formalism is most natural in the case of a relatively thick, distributed, gain region, in which case the standing wave factor is *ξ_g_* = 1. In the case of one or several QWs, when *d_g_ << L*, the notion of *g* is somewhat artificial, however, it can be introduced heuristically alongside a *ξ_g_* value of 1 < *ξ_g_* < 2 (see below). Using lumped gain per well, as in [19], is more rigorous (e.g., it gives *ξ_g_* > 1 self-consistently); however, it is also more complex, particularly in the case of multiple quantum well (QW) or quantum dot (QD) active layers separated by a substantial distance (e.g., located in different wave antinodes). The present formalism, in principle, applies to an arbitrary active layer thickness and location, though in this paper we shall concentrate on the most usual one, using a thin active layer in a single resonant location.

The usual differential equation for an injected laser (i.e., a vertical cavity amplifier with two strongly reflecting mirrors, operated above or near lasing threshold) is obtained by taking the absolute value of the denominator in Equation (1) to be small (which means operating above or near threshold, and simultaneously with a small frequency detuning from the cold cavity mode frequency). In the more general case of a resonator with arbitrary reflectances (in electrically pumped VECSELs, reflectances of 70–90% can be used [19,20]), we cannot assume the absolute value of the denominator in Equation (1) to be small; however, we can assume small frequency detuning: $\left|2\frac{nL}{c}{\tilde{G}}_{net}\Delta \omega \right|<<1$. Then,

(3) $$1-r_{i}r_{o}\tilde{G}e^{-j2\tilde{k}L}\approx 1-{\tilde{G}}_{net}+jT_{rt}{\tilde{G}}_{net}\Delta \omega ,$$

where:

(4) $${\tilde{G}}_{net}=\exp\left({\tilde{g}}_{net}L\right)=r_{i}r_{o}\exp\left[\left(\Gamma \tilde{g}-\alpha_{\mathrm{int}}\right)L\right]=\vartheta_{c}\tilde{G}$$

is the complex net round-trip gain, with:

(5) $$\vartheta_{c}=r_{i}r_{o}\exp\left(-\alpha_{\mathrm{int}}L\right)$$

the (real) cavity attenuation factor. We have also introduced the round-trip time of the cavity which, as usual in the theory of vertical cavity structures, is evaluated as $T_{rt}=2\frac{n_{g}L_{eff}}{c}$, where $n_{g}=n+\omega \frac{dn}{d\omega }$ is the group refractive index, and $L_{eff}=L+\frac{c}{n_{g}}\frac{d}{d\omega }\left(\left|\arg r_{o}+\arg r_{i}\right|\right)$ is the effective cavity length. Then, the resonator equation becomes:

(6) $$\left(1-{\tilde{G}}_{net}+jT_{rt}{\tilde{G}}_{net}\Delta \omega \right)E_{rc}=t_{i}^{}t_{i}^{\prime }r_{o}\tilde{G}e^{-j2\tilde{k}L}E_{inc}.$$

The active layer in a VECSEL is always thin so $\left|\tilde{G}\right|$ is never high above one (indeed, the measured chip reflectance has been reported [29,30] as *R_g_ =* |*r_g_*|^2^ for *r*_i_ ≈ 0.9, and *R_g_* ≈ 1.55 for *r*_i_ ≈ 0.96 (notations as in Equation (1)), from which the value of |$\tilde{G}$*|*–1 can be estimated to be of the order of 10^−2^ at most, meaning it is safe to approximate in Equation (6):

$$\tilde{G}\approx 1+\delta \tilde{G},\;\delta \tilde{G}=\Gamma \tilde{g}L$$

Strictly speaking, the expression (6) includes the dispersion of both the VECSEL active subcavity and the complex gain $\tilde{G}=\tilde{G}(\Delta \omega )$. If (as is usually the case) the operating wavelength is near the gain peak, we can use a Lorentzian gain spectrum approximation with a width *Δω_g_*.

Then, assuming as usual $\Delta \omega <<\Delta \omega_{g}$, the usual substitution $j\Delta \omega \to \frac{d}{dt}$ gives a single differential equation for the determination of the field reflected from the cavity:

(7) $$\left(T_{rt}\vartheta_{c}+\frac{f_{g}\delta \tilde{G}}{\Delta \omega_{g}}\right)\frac{dE_{rc}}{dt}=\left[\delta \tilde{G}-\left(1-\vartheta_{c}\right)\right]E_{rc}+\;t_{i}^{}t_{i}^{\prime }r_{o}e^{-\alpha_{\mathrm{int}}L}E_{inc}$$

where $\delta \tilde{G}=\Gamma \tilde{g}L$ is evaluated at the reference frequency $\omega_{ref}$ and $f_{g}=\frac{1}{1+j(\omega_{ref}-\omega_{p})/\Delta \omega_{g}}$ represents the carrier density dependent detuning between $\omega_{ref}$ and the spectral peak $\omega_{p}$ of gain. In a QW active material, the carrier density dependence of the detuning is weaker than in bulk material, so we can take *f_g_* = 1 = const. The equation combines cavity selectivity and gain dispersion and thus can, in general, describe the performance of a gain chip with arbitrarily small or large reflectances. In electrically pumped VECSEL designs, however, typically |*r*_i_| > ~ 0.5 [19,30], so, for realistic semiconductor active media (*Δω_g_* *>* ~10^13^s^−1^) we can comfortably assume that the spectral selectivity of the cavity dominates over the gain dispersion:

$$T_{rt}\vartheta_{c}>>\frac{\Gamma gL}{\Delta \omega_{g}}$$

Thus in the first approximation, we can neglect gain dispersion and write the equation in the simple form:

(8) $$T_{rt}\vartheta_{c}\frac{dE_{rc}}{dt}=\left[\delta \tilde{G}-\left(1-\vartheta_{c}\right)\right]E_{rc}+\;t_{i}^{}t_{i}^{\prime }r_{o}e^{-\alpha_{\mathrm{int}}L}E_{inc}$$

Equations (7) and (8) are the main result of this section.

### 2.2. The Full Mode-Locked Laser Model: The Linear Cavity

To consider the full cavity, we need to recall that with a single incident and single reflected beam in the linear cavity,

(9) $$E_{r}^{\left(g\right)}=E_{rc}+r_{i}{}^{\prime }E_{inc}$$

and note that:

(10) $$E_{inc}=\frac{\sqrt{\gamma }}{w{}^{\prime }}E_{r}^{\left(a\right)}(t-T_{ext})$$

where *T_ext_= L_ext_/c* is half of the round-trip time of the external cavity, and $E_{ra}$ is the field reflected from the absorber chip, γ is the dimensionless attenuation between the chips (including the out-coupling, if it is located between the chips, and the attenuation in the contact layer of an electrically pumped VECSEL), defined for the intensity as usual, hence, the square root in the equation for the field amplitude. Technically speaking, the value needs to be complex, with the phase dependent on the sub-wavelength variation in the length *L_ext_* of the internal cavity; however, in the linear cavity this can be set to zero with no loss of generality. The dimensionless parameter *w’* >1 is the ratio of the beam cross-section diameters in the gain (broader) and absorber (tighter to ensure saturation).

The absorber chip itself in the linear cavity can be described as in [19], by equations symmetric to those of the gain chip, with the incident field given by:

(11) $$E_{inc}^{\left(a\right)}=\sqrt{\gamma }w{}^{\prime }E_{r}^{\left(g\right)}(t-T_{ext})$$

(12) $$E_{r}^{\left(a\right)}=r{}^{\prime }{}_{ia}E_{inc}^{\left(a\right)}+E_{rc}^{\left(a\right)}.$$

Here, the field reflected from the inside of the chip is, assuming a (detuned) Lorentzian absorption spectrum, evaluated using an equation formally identical to (7):

(13) $$\left(T_{rt}^{\left(a\right)}\vartheta_{ca}-\frac{f_{a}\delta \tilde{A}}{\Delta \omega_{a}}\right)\frac{dE_{rc}^{\left(a\right)}}{dt}=-\left[\delta \tilde{A}+1-\vartheta_{ca}\right]E_{rc}^{\left(a\right)}+\;t_{ia}^{}t{}^{\prime }{}_{ia}^{}r_{oa}e^{-\alpha_{\mathrm{int},a}L_{a}}E_{inc}^{\left(a\right)}$$

As in the case of the gain chip,$\vartheta_{ca}=r_{ia}r_{oa}\exp\left(-\alpha_{\mathrm{int}}L\right)$, *r_0a_* and *r_ia_* being the outer and inner reflectances of the absorber cavity, $T_{rt}^{\left(a\right)}$ is the Saturable Absorber (SA) intrinsic cavity round-trip time, and the absorption in the cavity, evaluated at *ω*_ref_, at is quantified as:

(14) $$\;\delta \tilde{A}=\Gamma_{a}\tilde{\alpha }L_{a}.$$

The detuning factor $f_{a}=\frac{1}{1+j(\omega_{ref}-\omega_{pa})/\Delta \omega_{a}}$ needs to take into account that the SA is typically operating at the spectral slope of the absorber ($\omega_{ref}-\omega_{pa})/\Delta \omega_{a}$~1.

The complex gain and absorption in the case of bulk or QW active layers (QD layers may need more complex analysis) can be parametrized, using the phenomenological concepts of gain and absorption compression coefficients $\varepsilon_{g}$, $\varepsilon_{a}$, and linewidth enhancement factors for the carrier-dependent gain and absorption $\alpha_{H},\alpha_{Ha}$ and the nonlinearities $\alpha_{\varepsilon },\alpha_{\varepsilon a}$:

(15) $$\tilde{g}=g(N_{g})\left(\frac{1}{\left(1+\varepsilon_{g}S_{g}\right)}+j\left(\alpha_{H}-\alpha_{\varepsilon }\varepsilon_{g}S_{g}\right)\right)$$

(16) $$\tilde{\alpha }=\alpha (N_{a})\left(\frac{1}{\left(1+\varepsilon_{a}S_{a}\right)}+j\left(\alpha_{Ha}-\alpha_{\varepsilon a}\varepsilon_{a}S_{a}\right)\right)$$

where *S_g,a_* are the effective photon densities in the gain and absorption chip active layers. To write out the rate equations for carrier densities in both chips, we need the explicit expressions for these quantities, which depend on the geometry. Since $\left|\delta \tilde{G}\right|<<1,\;\left|\delta \tilde{A}\right|<<1$, we can estimate the field in the active layer of both chips in the passive resonator approximation. In the case of active layers in the form of (single or multiple) thin (QW or QD) layers very near to the field antinodes, which we shall follow in the analysis below, we can write:

(17) $$S_{g}={\left|{\bar{E}}_{\mathrm{int}}^{\left(g\right)}\right|}^{2},\;S_{a}={\left|{\bar{E}}_{\mathrm{int}}^{\left(a\right)}\right|}^{2},$$

where the fields inside the gain and SA active layers are evaluated assuming a resonant QW position as:

(18) $$E_{\mathrm{int}}^{\left(g\right)}\approx \frac{1+r_{o}}{t_{i}^{}r_{0}}E_{rc}^{\left(g\right)};\;E_{\mathrm{int}}^{\left(a\right)}\approx \frac{1+r_{oa}}{t_{ia}^{}r_{oa}}E_{rc}^{\left(a\right)}$$

From these expressions, the enhancement factors can be evaluated as:

(19) $$\xi_{}^{\left(g\right)}\approx \frac{{\left|1+r_{o}\right|}^{2}}{1+{\left|r_{0}\right|}^{2}};\;\xi_{}^{\left(a\right)}\approx \frac{{\left|1+r_{oa}\right|}^{2}}{1+{\left|r_{0a}\right|}^{2}}$$

which in the case of |$r_{o}$|~1 gives *ξ^(g)^* ≈ 2, the well-known result for the wave enhancement factor in an isolated VECSEL cavity with the QW active layer at the resonant position.

In the case of distributed (multilayer of bulk) gain or absorbing layers occupying a substantial fraction of the cavity, the enhancement factors *ξ* could be taken as *ξ* ≈ 1, with the intensities calculated as:

$$S_{g}\approx \left(1+{\left|r_{o}\right|}^{2}\right){\left|E_{rc}^{\left(g\right)}/t_{i}\right|}^{2},\;S_{a}\approx \left(1+{\left|r_{oa}\right|}^{2}\right){\left|E_{rc}^{\left(a\right)}/t_{ia}\right|}^{2}\;;\;\mathrm{however},\;\mathrm{that}\;\mathrm{case}\;\mathrm{is}\;\mathrm{not}\;\mathrm{considered}\;\mathrm{here}.\;$$

The effective photon densities *S_g,a_* then are used in the carrier rate equations:

(20) $$\frac{dN_{g}}{dt}=\frac{j}{ed_{g}}-\frac{N_{g}}{\tau_{g}(N_{g})}-\frac{v_{g}g(N_{g})}{1+\varepsilon_{g}S_{g}}S_{g}$$

(21) $$\frac{dN_{a}}{dt}=-\frac{N_{a}}{\tau_{a}(V_{a})}+\frac{v_{g}\alpha_{p}(N_{a})}{1+\varepsilon_{a}S_{a}}S_{a},$$

where as usual $v_{g}$= *c/n_g_*, $\tau_{a}(V_{a})$ is the absorber recovery time, *V_a_* being the (reverse) bias applied to the chip, if any. For the carrier dependences of gain and absorption, in this paper we use the standard phenomenological expressions:

(22) $$g_{p}(N_{g})=G_{0}\ln\frac{N_{g}+N_{s}}{N_{tr}+N_{s}}$$

(23) $$\alpha_{p}(N_{a})=\alpha_{0}-\sigma N_{a}$$

The dependences of gain and absorption bandwidths (BW) on respective carrier densities are estimated in the first approximation as linear, i.e.,

$$\Delta \omega_{g}(N_{g})=\frac{d\Delta \omega_{g}}{dN_{g}}(N_{g}-N_{g0}),$$

$$\Delta \omega_{a}(N_{a})=\Delta \omega_{a0}+\frac{d\Delta \omega_{a}}{dN_{a}}N_{a}.$$

### 2.3. The Main “Observable” Parameters

It is useful to establish the relations between parameters used in the model and the measurable values typically quoted in experiment, such as the threshold of the laser operation, the saturation fluence of the absorber, and the modulation contrast of the SA chip.

The threshold condition of the compound cavity is given by a transcendental equation, which in our notations is written as:

(24) $$\left(\frac{t_{i}^{2}r_{o}{\tilde{G}}_{th}\;e^{-j2\tilde{k}L}}{1-{\tilde{G}}_{th}\;r_{i}r_{o}e^{-j2\tilde{k}L}}+r_{i}{}^{{}^{\prime }}\right)\left(\frac{t_{ia}^{2}r_{oa}{\tilde{A}}_{0}\;e^{-j2\tilde{k}L_{a}}}{1-{\tilde{A}}_{0}r_{ia}r_{oa}e^{-j2\tilde{k}L_{a}}}+r_{ia}{}^{\prime }\right)\gamma e^{-j2k_{0}L_{inrercavity}}=1$$

where ${\tilde{G}}_{th}=1+\delta {\tilde{G}}_{th}$ and ${\tilde{A}}_{0}=1-\delta {\tilde{A}}_{0}$ are (complex) threshold gain and unsaturated absorption, and $k_{0}^{}=k_{ref}+\frac{\Delta \omega }{c}$ is the wave vector in a vacuum at the resonant (modal) frequency. The threshold condition is taken as the lowest gain of all the multiple solutions of the transcendental Equation (24), which corresponds to modes of the compound cavity. The numerical solution of essentially the same problem, though in different notations, illustrates [19] that, since the cavity length $L_{inrercavity}$>> *L, L_a_*, the modes are spaced closely enough for there always to be a few near the resonance of both chip resonators, essentially allowing us to count $e^{-j2k_{0}L_{inrercavity}}\approx 1$. In that case, assuming that the gain chip and SESAM are resonant cavities, we can establish an analytical estimate for the threshold in the form:

(25) $$g_{th}=\frac{1}{\Gamma L}\ln G_{th}\approx \frac{1}{\Gamma L}\left(G_{th}-1\right)\approx \frac{1}{\Gamma L}\left(\frac{\left(1+r_{i}r_{oA}\gamma \right)\left(1+\alpha_{\mathrm{int}}L\right)}{\left(r_{oA}\gamma +r_{i}\right)r_{o}}-1\right)$$

where:

(26) $$r_{oA}=\left|\frac{t_{ia}^{2}\vartheta_{ca}\left(1-\Gamma_{a}a_{0}f_{la}L_{a}\right)}{r_{i}\left(1-\vartheta_{ca}\left(1-\Gamma_{a}a_{0}f_{la}L_{a}\right)\right)}+r_{ia}{}^{\prime }\right|$$

is the unsaturated SA chip reflectance.

The saturation fluence of the absorber in the model we use (Equation (23)) is:

(27) $$F_{sat}\approx \frac{\hbar \omega }{\sigma }{\left|\frac{1-\left|{\tilde{A}}_{0}r_{ia}r_{oa}\right|}{t_{ia}^{}\left(1+r_{oa}\right)}\right|}^{2}$$

The reflectance contrast is estimated most easily by neglecting the self-phase modulation in the SA (since the Henry factor in the absorber is usually believed to be smaller than in the amplifier), and assuming small detuning from resonance, in which case:

(28) $$\Delta R\approx 2\left|r_{sA}\right|\left(\left|r_{sA}\right|-\left|r_{oA}\right|\right)\approx 2\left|r_{sA}\right|\frac{t_{ia}^{2}\vartheta_{ca}}{r_{ia}{\left(1-\vartheta_{ca}\right)}^{2}}\Gamma_{a}a_{0}L_{a}$$

where:

(29) $$r_{sA}=\frac{t_{ia}^{2}\vartheta_{ca}}{r_{i}\left(1-\vartheta_{ca}\right)}+r_{ia}{}^{\prime }$$

is the amplitude reflectance of a fully saturated absorber.

### 2.4. Numerical Results

The gain chip and the saturable-absorber parameters used in this section, unless specified otherwise, are listed in Table 1 and Table 2, respectively.

The values of the external cavity parameters, unless otherwise specified, are: the time of flight between the gain and the absorber cavities τ = 0.02 ns, the transmission coefficient *γ* = 1, and the ratio of the beam cross-section diameters onto the gain and the absorber chips *ω*′ = 3.

**Figure 2:**
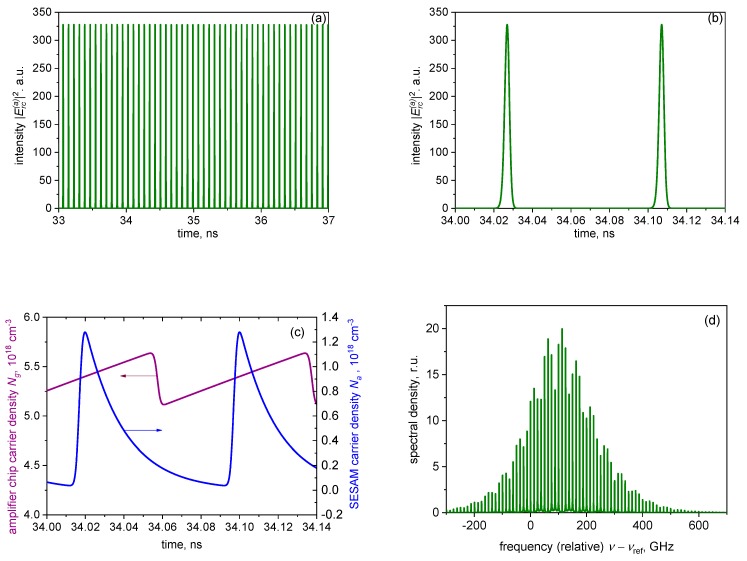
Time traces of the long-term (**a**) and short-term (**b**) evolution of the photon density reflected from the SESAM chip; (**c**) the corresponding evolution of the carrier densities; (**d**) the spectrum of (a). Gain chip current *i_c_* = 0.6 mA (stable mode locking).

**Figure 3:**
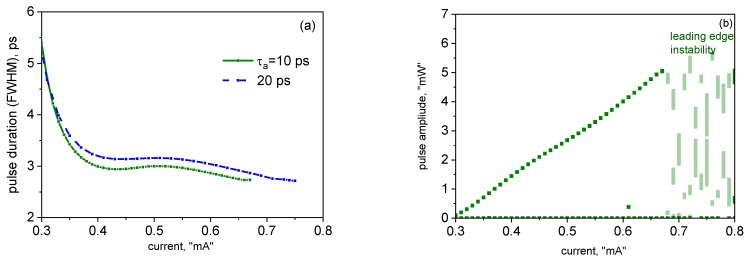
Current dependences of the pulse duration; (**a**) and amplitude (**b**). In (b), the absorber recovery time is 10 ps.

**Figure 4:**
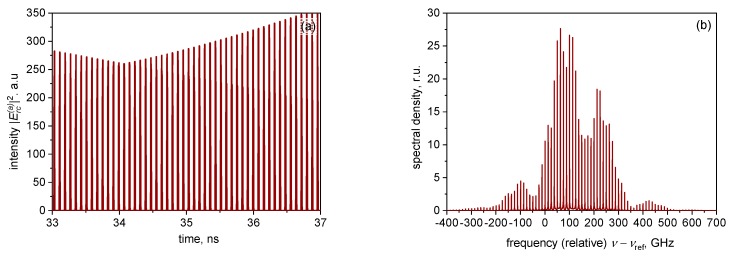
Time traces of the long-term evolution of the photon density reflected from the SESAM chip (**a**), and the corresponding spectrum (**b**). Gain chip current *i_c_* = 1.2 mA (unstable operation).

Figure 2, Figure 3 and Figure 4 present an example of simulated mode-locked operation of a VECSEL–SESAM configuration. Long- and short-term time traces of photon density $|E_{rc}^{\left(a\right)}{|}^{2}$ are shown in Figure 2a,b, respectively. Figure 2c shows the transient of carrier densities *N_a_* and *N_g_* corresponding to the photon density transient of Figure 2b. The optical spectrum of the time trace of Figure 2a is shown in Figure 2c.

As the modification of the pulse by a single round-trip is only moderate in the ML VECSEL, the pulse shape is fairly symmetric; however, the up-chirp is as usual in passively mode-locked semiconductor lasers still present, if relatively modest, with the time-bandwidth product of ΔνΔτ ≈ 0.6 (the pulse duration Δτ and the spectral width Δν being evaluated at half maximum), at the current shown. The chirp also manifests itself in the asymmetry, and in some envelope modulation of the spectrum.

The evolution of the pulse duration and amplitude with the current is illustrated in Figure 3a,b, respectively. The pulse duration is in the picosecond range and, as in [19], decreases overall with the current. As normal in mode-locked semiconductor lasers (see [31,32] and references therein), the pulses become longer with an increase in the absorber relaxation time.

As in [19], no trace of the “trailing edge” self-pulsing instability was observed in our simulations; this can be attributed to both the relatively low-repetition frequency and the weak pulse modification per pulse. However, at high currents, the irregular “leading edge” instability, in the form of two or, at higher currents, several non-periodically competing pulse trails (Figure 4a), is present; its onset has been chosen as the upper extent of the curves in Figure 3a.

As in edge-emitting ML lasers, the spectral signature of this unstable regime is the spectral shape (Figure 4b), which is less regular and with more envelope modulation than the spectrum of stable ML (Figure 2b).

## 3. VECSEL–SESAM in a Folded Geometry

### 3.1. Formulation of Delay-Differential Model for the Folded Cavity

**Figure 5:**
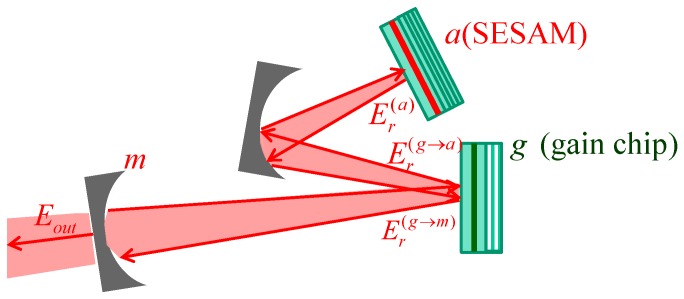
Mode-locked VECSEL–SESAM in folded geometry with an additional mirror.

In this section, we consider the case of a geometry that is alternative to the linear one treated in [19], and the previous section: The folded geometry. In this case, the three “reference points” of the cavity (Figure 5) are the output mirror (*m*), the gain chip (*g*), and the SESAM (*a*).

The purpose of the intermediate fully reflecting mirror is essentially for establishing the correct value of the width ratio *w′*.

In the folded cavity designs realized so far [21], it is the gain chip that is located in the middle of the cavity (Figure 5). In this case, the equation for the SESAM chip remains the same as the Equation (13), $T_{rt}^{\left(a\right)}\vartheta_{ca}\frac{dE_{rc}^{\left(a\right)}}{dt}=-\left[\delta \tilde{A}+1-\vartheta_{ca}\right]E_{rc}^{\left(a\right)}+\;t_{ia}^{}t^{\prime }{}_{ia}^{}r_{oa}e^{-\alpha_{\mathrm{int},a}L_{a}}E_{inc}^{\left(a\right)}$, and we still have:

(30) $$E_{inc}^{\left(a\right)}=\sqrt{\gamma }w\prime E_{r}^{\left(g\to a\right)}(t-T_{a-g})$$

where, *T_a-g_= L_a-g_/c* is the flight time between the gain and absorber chips, and $E_{r}^{\left(g\to a\right)}$ is the field reflected from the absorber chip in the direction of the gain chip.

For the gain section, the equation is functionally different, taking into account reflections in two directions. For the field leaving the cavity toward the output mirror, we would have:

(31) $$T_{rt}\vartheta_{c}\frac{dE_{rc}^{\left(g\to m\right)}}{dt}=\left[\delta \tilde{G}+\vartheta_{c}-1\right]E_{rc}^{\left(g\to m\right)}+\;t_{i}^{}t{}^{\prime }{}_{i}^{}r_{o}e^{-\alpha_{\mathrm{int}}L}E_{inc}^{\left(a\to g\right)}$$

The total field measured near the gain chip and propagating toward the output mirror then is:

(32) $$E_{r}^{\left(g\to m\right)}=r{}^{\prime }{}_{ia}E_{inc}^{\left(a\to g\right)}+E_{rc}^{\left(g\to m\right)}$$

For the field leaving the cavity toward the SESAM, we have:

(33) $$T_{rt}\vartheta_{c}\frac{dE_{rc}^{\left(g\to a\right)}}{dt}=\left[\delta \tilde{G}+\vartheta_{c}-1\right]E_{rc}^{\left(g\to a\right)}+\;t_{i}^{}t{}^{\prime }{}_{i}^{}r_{o}e^{-\alpha_{\mathrm{int}}L}E_{inc}^{\left(m\to g\right)}$$

The total field travelling from the VECSEL gain chip toward the SESAM then is:

(34) $$E_{r}^{\left(g\to a\right)}=r{}^{\prime }{}_{i}E_{inc}^{\left(m\to g\right)}+E_{rc}^{\left(g\to a\right)}$$

Finally, the field returning to the gain chip from the mirror is:

(35) $$E_{inc}^{\left(m\to g\right)}=r_{m}\gamma_{m}e^{j\Delta \varphi_{g-m}}E_{r}^{\left(g\to m\right)}(t-2T_{g-m})$$

with *T_g-m_* as the flight time between the gain chip and the mirror, and the factor $e^{j\Delta \varphi_{g-m}}$ taking into account the wavelength-scale cavity length variation. The field returning to the gain section from the SESAM will be the same as in the linear cavity:

(36) $$E_{inc}^{\left(a\to g\right)}=\frac{\sqrt{\gamma_{g}}}{w^{\prime }}E_{r}^{\left(a\right)}(t-T_{g-a})$$

The output field at the time *t* then is given by:

(37) $$E_{out}^{}=t_{m}\sqrt{\gamma_{m}}E_{r}^{\left(g\to m\right)}(t-T_{g-m}),t_{m}=\sqrt{1-r_{m}^{2}}$$

For the carriers in the VECSEL gain cavity we have the rate equation identical to (20); however, the intensity in the cavity is now due to propagation in both directions. In the plane wave approximation, and, as in [23,24], assuming in this study the incoherent addition of the counter propagating signals (applicable given a wide enough aperture), then the intensity within a thin resonantly positioned gain layer is:

(38) $$S_{g}\approx {\left|\frac{1+r_{o}}{t_{i}^{}}\right|}^{2}\left({\left|E_{rc}^{\left(g\to a\right)}\right|}^{2}+{\left|E_{rc}^{\left(g\to m\right)}\right|}^{2}\right)$$

### 3.2. Results of the Simulations for the Folded Cavity

**Figure 6:**
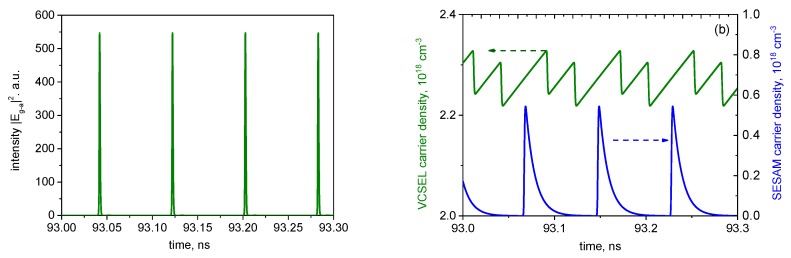
Dynamics of the photon (**a**) and carrier (**b**) densities in a short folded cavity.

Figure 6 illustrates the dynamics of the photon (a) and carrier (b) densities in a short folded cavity, with $T_{g-a}+T_{m-g}=\;40\;\mathrm{ps}$, corresponding to the repetition rate of ≈12.5 GHz (in the example shown, $T_{g-a}=\;25\;\mathrm{ps}$, $T_{m-g}=\;15\;\mathrm{ps}$). As in the case of the linear cavity, there is only one pulse in the cavity per round-trip; however the pulse is amplified in the gain chip twice per round-trip, which thus has a substantially shorter time to recover than in a linear cavity with the same overall length.

**Figure 7:**
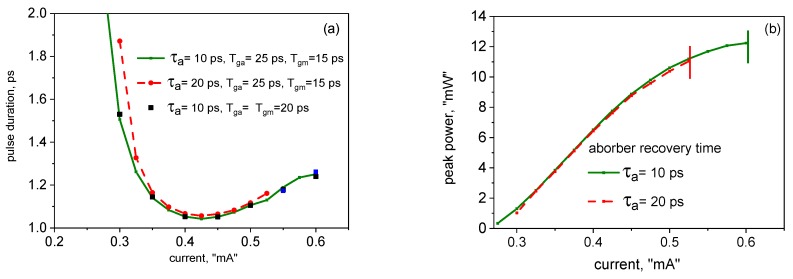
Current dependences of the pulse duration (**a**), and amplitude (**b**), in a short folded cavity.

Figure 7 shows the pulse duration and amplitude for the case of a short folded cavity as functions of the gain chip current. As in the linear cavity, and as is typical in all mode-locked semiconductor lasers, the pulse duration is somewhat longer for longer absorber recovery time. The current dependence of the pulse duration in this case is non-monotonic, decreasing with current at lower currents, as predicted also by [19] as well as by early generic theories of ML lasers with weak pulse modification per pulse (see e.g., [33]); however, increasing at higher currents, when pulse modification per pulse is more significant, in common with most edge-emitting ML lasers [31,32]. As in Figure 3, the upper limit of the curves is set by the onset of leading-edge chaotic instability.

**Figure 8:**
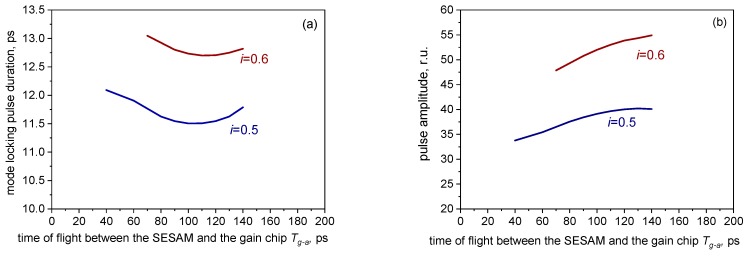
Dependences of the pulse duration (**a**), and amplitude (**b**), in a long folded cavity ($T_{g-a}+T_{m-g}=\;200\;\mathrm{ps}$) for two values of current.

For the relatively high-repetition rate shown in Figure 6 and Figure 7, the pulse parameters show almost no dependence on the relative length of the two branches of the cavity, so long as $T_{g-a}+T_{m-g}$ is kept constant (note the rectangular dots in Figure 7a). This is understandable, because, given $T_{g-a},T_{m-g}<<\tau_{g}$, the recovery of the population inversion in the gain chip (which is, strictly speaking, exponential) is virtually linear, and so the total depletion of the gain chip by both pulses does not depend on the relative magnitudes of the flight times $T_{g-a},T_{m-g}$. This dependence becomes more pronounced in longer cavities, when the flight times approach $\tau_{g}$ by an order of magnitude. This is illustrated in Figure 8, calculated for $T_{g-a}+T_{m-g}=\;200\;\mathrm{ps}$, or the repetition rate of ≈2.5 GHz. As seen in Figure 8, there is an optimal relation of the cavity branch lengths, in this case yielding the shortest ML pulse width, which, at least for the values of reflectances studied, corresponds to the gain chip near the middle of the cavity.

At relatively high currents, close to the onset of the leading edge instability, the geometry affects the stability limits: With the lengths of the branches strongly unbalanced, the gain chip current limit of stability is lower (the extent of the curves in Figure 8 corresponds to the stable single-pulse emission limit).

## 4. Colliding-Pulse Mode-Locking Configuration

### 4.1. Time-Delayed Model

**Figure 9:**
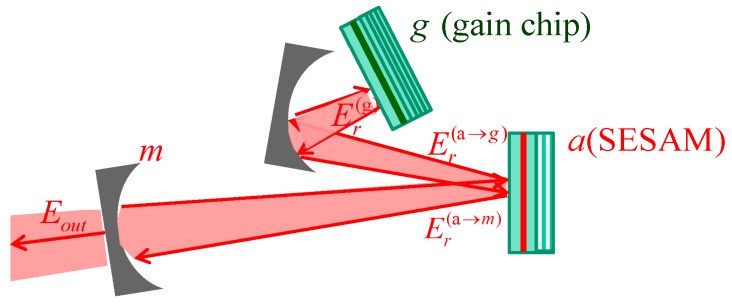
Colliding-pulse mode-locking folded geometry.

Lastly, we consider an alternative, and so far hypothetical, case (Figure 9) of the central chip being the SESAM, with the gain chip and the mirror *m* terminating the cavity, which is more difficult to realize (and which has not, to the best of our knowledge, been realized in this form so far); however, this alternative offers greater functionality: Potentially offering a colliding-pulse mode-locking (CPM) option. The equation system for this case is obtained from the equation for the folded cavity, with the gain chip in the middle, by the simple permutation of the symbols *g* and *a* in the notations.

Indeed, in a folded cavity with the SESAM in the “middle”, the equation for the gain chip remains the same as Equation (8):

$$T_{rt}\vartheta_{c}\frac{dE_{rc}}{dt}=\left[\delta \tilde{G}-\left(1-\vartheta_{c}\right)\right]E_{rc}+\;t_{i}^{}t_{i}^{\prime }r_{o}e^{-\alpha_{\mathrm{int}}L}E_{inc}$$

and we still have:

(39) $$E_{inc}=\frac{\sqrt{\gamma }}{w{}^{\prime }}E_{r}^{\left(a\to g\right)}(t-T_{a-g})$$

where *T_a-g_= L_a-g_/c* is the flight time between the gain and absorber chips, and $E_{r}^{\left(a\to g\right)}$ is the field reflected from the absorber chip in the direction of the gain chip.

For the SESAM, the equation is functionally different, taking into account reflections in two directions. For the field leaving the cavity toward the output mirror, we would have:

(40) $$T_{rt}^{\left(a\right)}\vartheta_{ca}\frac{dE_{rc}^{\left(a\to m\right)}}{dt}=-\left[\delta \tilde{A}+1-\vartheta_{ca}\right]E_{rc}^{\left(a\to m\right)}+\;t_{ia}^{}t\prime_{ia}^{}r_{oa}e^{-\alpha_{\mathrm{int}}L}E_{inc}^{\left(g\to a\right)}.$$

The total field toward the output mirror then is:

(41) $$E_{r}^{\left(a\to m\right)}=r\prime_{ia}E_{inc}^{\left(g\to a\right)}+E_{rc}^{\left(a\to m\right)}$$

For the field leaving the cavity toward the gain (VECSEL) chip, we have:

(42) $$T_{rt}^{\left(a\right)}\vartheta_{ca}\frac{dE_{rc}^{\left(a\to g\right)}}{dt}=-\left[\vartheta_{ca}\delta \tilde{A}+1-\vartheta_{ca}\right]E_{rc}^{\left(a\to g\right)}+\;t_{ia}^{}t{}^{\prime }{}_{ia}^{}r_{oa}e^{-\alpha_{\mathrm{int}}L}E_{inc}^{\left(m\to a\right)}.$$

The total field travelling from the SESAM toward the VECSEL chip then is:

(43) $$E_{r}^{\left(a\to g\right)}=r{}^{\prime }{}_{ia}E_{inc}^{\left(m\to a\right)}+E_{rc}^{\left(a\to g\right)}.$$

Finally, the field returning to the SESAM from the mirror is:

(44) $$E_{inc}^{\left(m\to a\right)}=r_{m}\gamma_{m}e^{j\Delta \varphi_{a-m}}E_{r}^{\left(a\to m\right)}(t-2T_{a-m})$$

whereas the field returning to the SESAM from the gain section will be the same as in a linear cavity:

(45) $$E_{inc}^{\left(g\to a\right)}=\sqrt{\gamma_{g}}w{}^{\prime }E_{r}^{\left(g\right)}(t-T_{g-a}).$$

The output field at the time *t* then is given by:

(46) $$E_{out}^{}=t_{m}\sqrt{\gamma_{m}}E_{r}^{\left(a\to m\right)}(t-T_{a-m}),t_{m}=\sqrt{1-r_{m}^{2}}$$

Inside the SESAM cavity we still have the same rate equation; however, the fields exist due to propagation in both directions. In the plane wave approximation, and with a thin resonantly positioned absorber,

(47) $$S_{a}\approx {\left|\frac{1+r_{oa}}{t_{ia}^{}}\right|}^{2}\left({\left|E_{rc}^{\left(a\to g\right)}\right|}^{2}+{\left|E_{rc}^{\left(a\to m\right)}\right|}^{2}\right)$$

The absorber saturation fluence in the folded cavity, with either absorber position, would be the same as in the linear geometry (though effectively in the colliding-pulse design it will become twice smaller, with two pulses arriving simultaneously), and the threshold condition in the CPM cavity becomes:

(48) $$g_{th}\approx \frac{1}{\Gamma L}\left(G_{th}-1\right)\approx \frac{1}{\Gamma L}\left(\frac{\left(1+r_{i}r_{m}r_{oA}^{2}\gamma \gamma_{m}^{}\right)\left(1+\alpha_{\mathrm{int}}L\right)}{\left(r_{m}r_{oA}^{2}\gamma \gamma_{m}^{}+r_{i}\right)r_{o}}-1\right).$$

### 4.2. Calculations and Results

**Figure 10:**
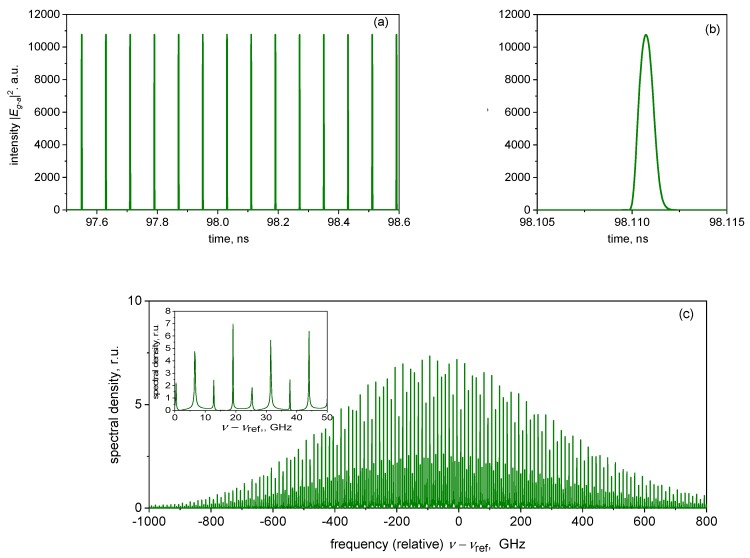
Mode-locked VECSEL–SESAM-output mirror structure time traces of $|E_{g-a}^{}{|}^{2}$: (**a**) pulse trail, and; (**b**) single pulse; (**c**) shows the spectrum of the time trace of (**a**), with a fragment in an inset. Injection current is *j* = 1.1 mA; absorber recovery time τ_a_ = 10 ps.

Figure 10 presents the schematic of evolution of the output photon density in a CPM configuration (a,b), and the corresponding spectrum (c), for one operating current in a short ($T_{g-a}=T_{m-g}=\;40\;\mathrm{ps}$) cavity. The time-domain pulse trail (Figure 10a) shows complete repetition frequency doubling when compared to a linear cavity (two pulses per round-trip; note the identical amplitudes of adjacent pulses and the repetition period, the same as in Figure 2, despite a twice longer cavity), as expected for well-developed CPM operation. However, in the spectrum, the doubling of frequency intervals between modes is not complete; intermediate modes corresponding to the round-trip of the entire cavity are somewhat suppressed, yet still present (see inset to Figure 10c). Similar performance was simulated for edge-emitting mode-locked lasers under certain conditions [34].

**Figure 11:**
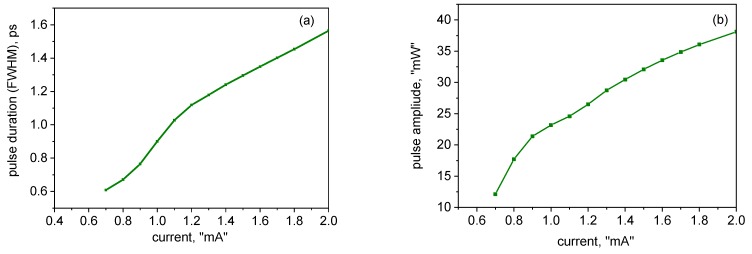
Current dependences of the pulse duration (**a**), and amplitude (**b**), in the CPM design. *τ_a_* = 10 ps.

Figure 11 shows the evolution of the CPM pulse duration and amplitude with the current. In this geometry, the simulated pulsewidth increases with the current through the current range studied; as mentioned above, this is typical for edge-emitting lasers, and can be associated with relatively strong modification of the pulse per round-trip. In edge emitters, such a situation is associated with an asymmetric shape with the longer trailing edge, which indeed is observed also in our simulations (Figure 10b).

The upper extent of the curve, as in Figure 3 and Figure 7, is the onset of the leading edge instability. The stability range for this design, with two pulses saturating the absorber simultaneously, is substantially higher than in the case of a simple linear cavity with the same repetition rate, and the pulses, shorter, by virtue of more efficient absorber saturation, which is one of advantages of CPM [31,32,34]. The cavity thus looks suitable, in principle, for picosecond pulse generation. In the case of the femtosecond regime using a low-dispersion optically pumped active chip, the more complex, yet also more reliable ring CPM geometry used in recent studies [35], may be preferable (the folded cavity studied here would need micrometer-scale balancing of subcavity lengths). More detailed investigation is reserved for future work.

## 5. Conclusions

We have presented a simple, versatile model for the dynamics of electrically and optically pumped vertical-external-cavity surface-emitting lasers mode locked by a semiconductor saturable-absorber mirror. Time delays in the external cavities formed by the VECSEL gain chip and the saturable-absorber mirror and output mirror are accounted for. Analytical expressions for the experimentally accessible characteristics of the system are provided, namely, the threshold gain and saturation fluence and reflection contrast of the absorber. For realistic parameters of the semiconductor cavities, the model predicts fundamental mode locking with ps pulse duration. The dependences of the pulse width and pulse amplitude, as well as the frequency chirp are investigated as a function of the injection current. The model is easily generalized for different VECSEL and SA configurations, and examples for the case of folded geometry with the central chip being either the gain section or the SESAM are presented. Future work can concentrate on perfecting the model for the folded cavities, including an account of a partly coherent addition of signals (as in [35]) and possibly the lateral effects, as well as the polarization properties and more diverse geometries.

## Figures and Tables

**Table 1:** Gain chip parameters. QW: Quantum well.

Parameter	Notation	Value	Units
back mirror amplitude reflectivity	*r_o_*	0.9995	
front mirror amplitude reflectivity	*r_i_*	0.7	
internal losses	*α_i_*	0.001	μm^−1^
effective length	*L_a_*	1.5	μm
QWs cumulative thickness	*d_g_*	0.024	μm
radius of the active region	*ρ_g_*	3	μm
group refractive index	*n_rg_*	3.5	
confinement factor	*Γ_g_*	0.06	
linewidth enhancement factor	*α_g_*	3	
carriers lifetime	*τ_g_*	1	ns
gain compression factor	*ε_g_*	0.5 × 10^−5^	μm^−3^
parameter carrier density	*N_s_*	−0.4 × 10^6^	μm^−3^
transparency carrier density	*N_tr_*	1.6 × 10^6^	μm^−3^
gain coefficient	*G_0_*	0.18	μm^−1^
coefficients of gain bandwidth dependence	*dΔω_g_/dN_g_* *N_g0_*	0.012 7.5 × 10^5^	μm^3^/ns μm^−3^

**Table 2:** Saturable-absorber parameters.

Parameter	Notation	Value	Units
back mirror amplitude reflectivity	*r_oa_*	0.97	
front mirror amplitude reflectivity	*r_ia_*	0.565	
internal losses	*α_ia_*	0.001	μm^−1^
effective length	*L_a_*	1.5	μm
group refractive index	*n_ra_*	3.5	
confinement factor	*Γ_a_*	0.06	
linewidth enhancement factor	*α_a_*	3	
carriers lifetime	*τ_a_*	0.03	ns
compression factor	*ε_a_*	1.5 × 10^−5^	μm^−3^
absorber saturation cross-section	*σ*	2 × 10^−7^	μm^−3^
absorption coefficient	*α_0_*	0.5	μm^−1^
coefficients of absorption bandwidth dependence	*dΔω_a_/dN_a_* *Δωa_0_*	0.048 15199	μm^3^/ns 1/ns
